# Increased Expression of Transferrin Receptor 1 in the Brain Cortex of 5xFAD Mouse Model of Alzheimer’s Disease Is Associated with Activation of HIF-1 Signaling Pathway

**DOI:** 10.1007/s12035-024-03990-3

**Published:** 2024-02-01

**Authors:** Sabrina Petralla, Liudmila Saveleva, Katja M. Kanninen, Julia S. Oster, Maria Panayotova, Gert Fricker, Elena Puris

**Affiliations:** 1 Institute of Pharmacy and Molecular Biotechnology, Ruprecht-Karls-University, Im Neuenheimer Feld 329, 69120 Heidelberg, Germany; 2 A.I. Virtanen Institute for Molecular Sciences, University of Eastern Finland, P.O. Box 1627, 70211 Kuopio, Finland

**Keywords:** Alzheimer’s disease, Transferrin receptor, HIF-1, 5xFAD mice, Brain microvessels, Brain cortex

## Abstract

Alzheimer’s disease (AD) is the most common cause of dementia. Despite intensive research efforts, there are currently no effective treatments to cure and prevent AD. There is growing evidence that dysregulation of iron homeostasis may contribute to the pathogenesis of AD. Given the important role of the transferrin receptor 1 (TfR1) in regulating iron distribution in the brain, as well as in the drug delivery, we investigated its expression in the brain cortex and isolated brain microvessels from female 8-month-old 5xFAD mice mimicking advanced stage of AD. Moreover, we explored the association between the TfR1 expression and the activation of the HIF-1 signaling pathway, as well as oxidative stress and inflammation in 5xFAD mice. Finally, we studied the impact of Aβ_1–40_ and Aβ_1–42_ on TfR1 expression in the brain endothelial cell line hCMEC/D3. In the present study, we revealed that an increase in TfR1 protein levels observed in the brain cortex of 5xFAD mice was associated with activation of the HIF-1 signaling pathway as well as accompanied by oxidative stress and inflammation. Interestingly, incubation of Aβ peptides in hCMEC/D3 cells did not affect the expression of TfR1, which supported our findings of unaltered TfR1 expression in the isolated brain microvessels in 5xFAD mice. In conclusion, the study provides important information about the expression of TfR1 in the 5xFAD mouse model and the potential role of HIF-1 signaling pathway in the regulation of TfR1 in AD, which could represent a promising strategy for the development of therapies for AD.

**Supplementary Information:**

The online version contains supplementary material available at 10.1007/s12035-024-03990-3.

## Introduction

Transferrin receptor 1 (TfR1, encoded by *TFRC*) is a key player in regulation of the brain distribution of iron, which is involved in several biological processes in the brain including cellular metabolism and proliferation during development as well as myelination and neurotransmission [1, 2]. TfR1 was reported to be expressed on both the apical and basal plasma membranes of the brain endothelial cells forming the blood–brain barrier (BBB), with apicobasal bi-directional transport capability [3, 4]. At the luminal side, TfR binds to an iron-laden transferrin (Tf) molecule and internalizes it via endocytosis. Upon maturation, the endosome is acidified, and iron is released from Tf and transported to the cytosol by a divalent metal transporter 1 (DMT1), while Tf and TfR are either recycled or transported back to the luminal membrane [5–7]. The Tf/TfR1/DMT1-mediated mechanism is the main iron uptake process not only in the brain endothelial cells, but also in neurons, astrocytes, and oligodendrocyte precursor cells [2, 8, 9]. In addition to its important physiological role, TfR1 expression in the brain endothelial cells makes it an attractive target for drug delivery to the brain [10, 11]. Several drug delivery strategies have been developed to target TfR1 at the BBB for delivery of drugs to the brain [7, 12, 13].

Changes in TfR1 expression and function can lead to consequent dysfunction in iron transport contributing to pathogenic processes in the brain, as well as alter the brain delivery of drugs targeting this receptor. One of the diseases characterized by iron dyshomeostasis and ferroptosis is Alzheimer’s disease (AD) [14, 15]. AD is the most common cause of dementia, with the major hallmarks of accumulated fibrillar and amorphous amyloid-β peptide (Aβ) aggregates and intracellular neuronal aggregates of hyperphosphorylated τ forming neurofibrillary tangles [16]. Despite intensive research and progress in developing treatments to change the disease progression, AD remains uncurable. In AD, metals, including iron, are involved in the polymerization of Aβ peptide as well as in mediating the neurotoxic action of Aβ protofibrils [17]. Studies in AD patients demonstrated that iron accumulates in the parietal cortex and hippocampus and this accumulation is connected to Aβ and τ pathologies [18, 19]. Moreover, iron homeostasis disorder leads to higher iron uptake and toxicity resulting in oxidative stress, ferroptosis, and neuronal loss, all of which are prominent characteristics of AD pathology [14, 20]. In this respect, TfR1, a major regulator of iron distribution to the brain and within the brain, can contribute to iron dyshomeostasis observed in AD.

The information about changes in expression of TfR1 in AD patients and animal models is limited and controversial to date. Recently, Bourassa et al. (2019) reported no differences in TfR1 levels in whole homogenates from post-mortem parietal cortex and hippocampus of AD patients as well as in isolated brain microvessels from parietal cortex [21]. Additionally, TfR1 levels were not changed in isolated brain microvessels of 12- and 18-month-old non-transgenic and 3xTg-AD mouse model of AD [21]. In another study, changes in the expression of TfR in AD brain were found to be region-specific [22]. Thus, in contrast to Bourassa et al. (2019), a reduction in TfR levels was found in the temporal and occipital cortices and the hippocampus of AD patients, while no differences in TfR levels were observed in the parietal and frontal cortical regions [22]. Importantly, studies in AD animal models showed that brain expression of TfR1 as well as its binding capacity can change with the course of AD progression and can be related to the production and accumulation of Aβ [5, 23]. Lu and colleagues suggested that TfR1 is upregulated at the early stage of AD in the cortex and hippocampus of APP/PS1 Tg mice, whereas the expression of TfR1 begins to decrease with disease progression [5, 24]. However, the effect of the presence of Aβ around the vasculature on TfR1/*TFRC* expression in the brain microvessels in AD has not been studied.

TfR1 expression has been shown to be regulated by the status and availability of iron: TfR1 is upregulated when iron status is low, and downregulated when iron status is high [2]. Under certain conditions such as oxidative stress, inflammation, and hypoxia, expression of hypoxia-inducible factors (HIF) is induced, and the binding of iron-regulatory proteins IRP1 and IRP2 can promote *TFRC* transcription [25–27]. Protein expression of Hypoxia-inducible factor 1-alpha (HIF1A) was increased in the App^NL−P−F/NL−P−F^ (NL-P-F) mouse model of AD [28]. The modulation of HIF-1 signaling pathway in glial cells in AD has been considered as a potential therapeutic approach [29, 30]. However, the involvement of this pathway in regulation of TfR1/*TFRC* expression in the AD brain has not been studied.

In the present study, we aimed to investigate whether the HIF-1 signaling pathway is involved in regulation of changes in TfR1 expression in the brain at the advanced stage of AD. To achieve this goal, we studied TfR1/*TFRC* expression in cortical samples and isolated brain microvessels from 8-month-old female 5xFAD mice as compared to age- and sex-matched wild-type animals. We also investigated the association between the observed changes in TfR1/*TFRC* expression and the oxidative state, inflammation, and hypoxia in the cortex. In addition, we investigated if the Aβ_1–40_ and Aβ_1–42_ peptides can affect expression of TfR1/*TFRC* in brain endothelial cells in vitro. For that purpose, we used the immortalized brain endothelial cell line, such as hCMEC/D3 [31], which is commonly used in vitro model in BBB research and has been shown to express TfR1 [32].

## Materials and Methods

### Materials

Guanidine hydrochloride (#G3272), acetonitrile (#1.00029), formic acid (5.33002), ethylenediaminetetraacetic acid (EDTA) (#E9884), Tris–HCl (#10,812,846,001), bovine serum albumin (BSA, A3294), NaCl (#S9888), KH_2_PO_4_ (#P5655), KCl (#P3911), HEPES (#H3375), CaCl_2_·2H_2_O (#223,506), MgSO_4_·7H_2_O (#1,374,361), dextran (#31,390), Sucrose (#S0389), sodium dodecyl sulfate (SDS; #151–21-3), bromophenol blue (#115–39-9), Tween-20 (#9005–64-5), and protease inhibitor cocktail (#11,836,170,001) were purchased from Sigma-Aldrich (St. Louis, MO). Aβ_1-40_ (#AS-24235) and Aβ_1-42_ (#AS-20276) peptides were purchased from (Eurogentec, Belgium). ProteoExtract® Subcellular Proteome Extraction Kit (#539,790), NP-40 (#492,016) were purchased from Merck KGaA, Darmstadt, Germany.

### Study Design and Experimental Model

The study was performed according to the Council of Europe Legislation and Regulation for Animal Protection, complied with the ARRIVE guidelines and EU Directive 2010/63/EU for animal experiments as a part of our previous study [33]. The Animal Experiment Board in Finland (Regional State Administrative Agency of Southern Finland) approved the use of animals in the study (licence number ESAVI-2018–012856). We used transgenic hemizygous 5xFAD mice (RRID:MMRRC_034848-JAX, Jackson Laboratories, Bar Harbor, ME, USA) with the *APP* Swedish, Florida, and London mutations in human *APP* as well as with the M146L and L286V mutations in human *PSEN1* driven by the mouse *Thy1* promoter [34] and their wild-type (WT) littermates on the C57BL/6 J background (RRID:IMSR_JAX:000664, Jackson Laboratories, Bar Harbor, ME, USA). Mouse genotyping was described previously [33]. We used only female animals in the study, as female 5xFAD mice develop AD-mimicking characteristics faster than male mice and due to a higher AD prevalence in women [35, 36]. We used 8-month-old mice due to the development of AD-relevant pathological features at this age, including extensive amyloid pathology, cognitive impairment, and neuroinflammation deficits of potentiation and synaptic transmission by this age [33, 37–40]. Mice were housed under standard conditions: controlled temperature (21 ± 1 °C) and humidity (50%), 12:12-h light–dark cycles, with access to water and maintenance diet ad libitum. The bedding material was aspen chips (Tapvei, Finland), while the nest material (aspen wool, Tapvei, Finland), plastic tube/iglu and nestlet dams were used as enrichment.

### Tissue Collection and Brain Microvessel Isolation

The animals were killed by means of carbon dioxide asphyxiation since anesthetics might affect the expression of transferrin receptor. The blood was removed by transcardial perfusion with heparinised 0.9% saline (2500 IU/L, LEO) and the mouse brains were dissected out of the skull, excised, followed by extraction of the cerebrums. The brain cortex was collected and immediately placed on ice cold Buffer 1 (101 mM NaCl, 1.2 mM KH_2_PO_4_, 4.6 mM KCl, 15 mM HEPES, 5 mM CaCl_2_·2H_2_O, 1.2 mM MgSO_4_·7H_2_O, pH 7.4) for microvessel isolation. A part of prefrontal cortex (ca. 15 mg) was snap frozen and stored at − 80 °C until the further analysis.

Mouse brain microvessels were immediately isolated using a combination of a dextran density gradient separation with size filtration according to the previously validated and applied protocol [33, 41]. The procedure was conducted at 4 °C. In brief, the combined mouse brain cortices (6–7 brain cortices to produce one brain microvessel sample) dissected into 1 mm pieces were mixed with Buffer 1 (5 volumes per gram of tissue weight), and homogenized by the Potter–Elvehjem homogenizer with 20 up-and-down, unrotated strokes. After that, the homogenates were centrifuged at 2000 × *g* for 10 min at 4 °C, and the pellet was suspended in Buffer 2 (Buffer 1 containing 16% dextran). The suspension was centrifuged at 4500 × *g* for 15 min at 4 °C. The supernatant was transferred to a new tube, and centrifuged again in a similar manner. Both produced pellets were combined after suspension in Buffer 3 (Buffer 1 containing 5 g/L BSA). In the next step, the suspension was passed through a pre-wet nylon mesh of 200 µm (PluriStrainer® 200 µm, #43–50,200-03, PluriSelect Life Science, Germany) followed by washing of the mesh with 10 mL of Buffer 3. The flow-through was passed through a pre-wet nylon mesh of 100 µm (PluriStrainer® 100 µm, #43–57,100-51, PluriSelect Life Science, Germany), followed by washing of the mesh with 10 mL of Buffer 3. The flow-through was loaded to a pre-wet nylon mesh of 20 µm (PluriStrainer® 20 µm, #43–50,020-03, PluriSelect Life Science, Germany), which was then washed with 40 mL of Buffer 3. As a result, the brain microvessels, which were retained on the mesh, were immediately collected by washing the mesh with 30 mL of Buffer 3 and centrifugation at 1000 × *g* for 5 min at 4 °C. The resulting pellet representing the isolated brain microvessels was suspended by adding 1 mL of Buffer 1 and centrifuged at 1000 × *g* for 5 min at 4 °C. The supernatant was removed, and the obtained pellet was used for isolation of subcellular fractions (crude membrane, cytosol, and nucleus) using ProteoExtract® Subcellular Proteome Extraction Kit (#539,790) according to the manufacturer’s instructions. The total protein levels were measured in the obtained fractions of the isolated cerebral microvessel samples using the Bio-Rad DC Protein Assay. The fractions were stored at − 80 °C until further analysis.

### Cell Culture and In Vitro Experiments with Aβ_1-40_ and Aβ_1-42_

Human brain capillary endothelial cell line (hCMEC/D3 cell, RRID:CVCL_U985) was kindly provided by Prof. Dr. Jörg Huwyler (University of Basel, Switzerland). The cells were seeded on collagen type I coated 10 cm dishes (density 1.5 × 10^6^ cells) for protein quantification or 6 well plates (density 0.3 × 10^6^ cells/well) for qRT-PCR analysis. For routine culture, cells were seeded in T-75 flasks (Corning, USA) with a seeding density of 0.1 × 10^6^ under standard conditions for 7 days. The cells were cultured with endothelial cell growth medium 2 (PromoCell GmbH, Heidelberg, Germany) supplemented with 0.02 mL/mL fetal calf serum, 10 ng/mL recombinant human basic fibroblast growth factor, 5 ng/mL recombinant human epidermal growth factor, 0.5 ng/mL recombinant human vascular endothelial growth factor 165, 10 ng/mL recombinant human basic fibroblast growth factor, 20 ng/mL insulin-like growth factor, 22.5 µg/mL heparin, 1 µg/mL ascorbic acid, 0.2 µg/mL hydrocortisone and antibiotics (1% penicillin–streptomycin) in an atmosphere of 95% air and 5% CO_2_ at 37 °C. The cell culture medium was changed every 2 days. The passage number was between 32 and 35.

On day 3, when the hCMEC/D3 cells reached 60% confluency, the medium containing 0.1 μM Aβ_1-40_ and Aβ_1-42_ was added to the cells in the treatment groups, while for the control group medium without Aβ peptides was used (*n* = 4 biological replicates per study group). After 48-h incubation (at confluency), the cells seeded to 10 cm dishes were washed and scraped for the crude membrane isolation described below, while cells cultured in 6 well plates were used for gene expression analysis described below.

### Cell Viability Assay

The effect of Aβ_1-40_ and Aβ_1-42_ on viability of hCMEC/D3 cells was PrestoBlue Assay Kit (Invitrogen, Carlsbad, CA, USA) as previously described [42]. Briefly, hCMEC/D3 cells were seeded on collagen type I coated 96-well plates (density 0.1 × 10^5^ cells). On third day of the experiment, cells were washed twice with Hanks’ Balanced Salt Solution supplemented with sodium pyruvate and HEPES buffer solution followed by addition of either 0.1 μM Aβ_1-40_ or Aβ_1-42_ or cell culture medium (control cells). The cells were incubated under standard conditions for 48 h. Untreated cells were used as negative controls with 100% viability. After incubation, the PrestoBlue™ Cell Viability Reagent was added, and fluorescence was measured using a fluorescence plate reader Infinite® P200 Pro (Tecan, Crailsdorf, Germany) with excitation and emission wavelengths of 560 nm and 590 nm.

### Quantitative Reverse Transcription Polymerase Chain Reaction (qRT-PCR)

In the present study, we quantified gene expression of *Tfrc*, hypoxia-inducible factor 1-alpha (*Hif1a*), a pro-inflammatory cytokine, interleukin-1 beta (*Il1b*) and an oxidative stress marker sirtuin-3 (*Sirt3*) by qRT-PCR analysis in brain cortices of WT and 5xFAD mice. In addition, gene expression of *TFRC* in hCMEC/D3 cells with and without Aβ_1-40_ or Aβ_1-42_ treatment was quantified. First, total RNA was extracted using RNeasy Mini Kit (#74,004, Qiagen, Stockach, Germany) from the samples according to the manufacturer’s instructions and quantified by NanoDrop (Thermo Scientific, Dreieich, Germany). Biozym cDNA synthesis Kit (#331475S, Oldendorf, Germany) was used for cDNA synthesis according to the manufacturer’s instructions. The obtained cDNA was mixed with gene-specific primers from ThermoFisher Scientific (Table S1 and S2) and the PowerUp ™ SYBR ™ Green Master Mix (#A25741, Thermo-Fischer, Waltham, USA). Relative target gene expression of mouse *Tfrc*, *Hif1a*, *Il1b*, and *Sirt3* in mouse brain cortices was normalized to the housekeeping gene, beta-actin (*Actb*), while relative target gene expression of *TFRC* in cells was normalized to the housekeeping gene, glyceraldehyde‐3‐phosphate dehydrogenase (*GAPDH*). Gene expression in each sample was estimated according to the method explained previously [43]. The qRT-PCR analysis was performed with LightCycler 96 (Roche Diagnostics). The data acquisition was performed with the LightCycler® 96 SW 1.1 software, v. 1.1.0.1320 (Roche Diagnostics, Mannheim, Germany; 2011).

### Western Blotting

Equal protein amounts from each sample were mixed with Laemli electrophoresis loading buffer (1 M Tris–HCl, pH 6.8; 20% SDS; 0.4 μL/mL glycerol; 2 g/L bromophenol blue and 2 M DTT) and resolved in 10–15% acrylamide gels through SDS-PAGE. Proteins were transferred into nitrocellulose membranes for 120 min at a constant current of 400 mA. Membranes were then blocked in PBS-0.1% Tween 20–5% skimmed milk for 1 h and then incubated overnight with primary antibodies against TfR1 (1:1000; Cat# NB100-92243, Novusbio), HIF1A (1:1000; Cat# sc-10790, SantaCruz Biotechnology), GAPDH (1:5000; Cat# NB300-327, Novusbio), histone H3 (1:1000; Cat# 9715, Cell Signaling) and mouse IC16 antibody recognizing residues 1–16 of the human Aβ (1:500; kindly provided by Prof. Claus U. Pietrzik) diluted in PBS-0.1% Tween 20–5% skimmed milk. Then, membranes were washed 3 × for 10 min with PBS-0.1% Tween 20 and incubated with HRP-linked secondary antibody goat anti-rabbit (1:5000; Cat# 111–035-144, Jackson ImmunoResearch Labs) and with HRP-linked secondary antibody goat anti-mouse (1:5000; Cat# 074–1806, KPL Kirkegaard & Perry Labs) for 90 min at RT and visualized by Western Lightning Plus- ECL (Enhanced ChemiLuminescence Substrate; PerkinElmer). Images were acquired with a Bio-Rad ChemiDoc imager and densitometric analysis was performed by using the Bio-Rad ImageLab software (Version 5.1).

### Measurement of Reactive Oxygen Species (ROS) Production

Mouse brain cortices were homogenized in isotonic buffer (10 mM Hepes, 200 mM mannitol, 70 mM sucrose, 1 mM EDTA pH 7.6, 1% NP40, 1X protease inhibitor cocktail) at a 1:10 tissue weight/lysis buffer volume ratio with a Potter homogenizer (30 strokes at 1000 rpm) while kept on ice. The total protein concentration was determined using Bio-Rad DC protein assay (Bio-Rad Laboratories, Hercules, CA, USA) according to the manufacturer’s recommendations. ROS production was measured in the homogenates at a final concentration of 0.2 µg total protein/µL using the ROS-ID® Total ROS/Superoxide Detection Kit (ENZ-51010, Enzo Life Sciences, Farmingdale, NY, USA). All samples were incubated with the ROS/Superoxide Detection Solution, containing 2 µM of the Oxidative Stress Detection Reagent and Superoxide Detection Reagent each for 60 min at 37 °C in the dark. Fluorescence was detected with a TECAN Infinite F200 Pro plate reader (Tecan Group, Männerdorf, Switzerland) using fluorescein (excitation at 485 nm, emission at 535 nm) and rhodamine filters (excitation at 540 nm, emission at 590 nm). Data are presented as percentage (%) of control.

### Statistical Analysis

This is an exploratory study. The normalized protein expression of TfR1 and HIF1A in the brain cortical tissue (*n* = 7 per study group) as well as in the isolated brain microvessels (*n* = 7 for WT and *n* = 4 for 5xFAD mice) of 5xFAD mice are presented as percentage of control WT mice (mean ± SEM). The normalized fold expression of *Tfrc*, *Hif1a*, *Il1b*, and *Sirt3* in the brain cortical tissue (*n* = 7–8 per group) of 5xFAD mice are presented as percentage of control WT mice (mean ± SEM). The normalized protein expression of TfR1 (*n* = 4 per group) and the normalized fold expression of *TFRC* (*n* = 6 per group) in the hCMEC/D3 treated with either Aβ_1-40_ and Aβ_1-42_ are presented as percentage of control non-treated group (mean ± SEM). Total ROS and superoxide production in the brain cortical tissue (*n* = 5 per study group) of 5xFAD mice are presented as percentage of control WT mice (mean ± SEM). Statistical significance of differences in normalized fold gene expression of *Tfrc*, *Hif1a*, *Il1b*, and *Sirt3*, protein expression of TfR1 and HIF1A and total ROS and superoxide production between study groups was analyzed using an unpaired *t*-test. A *p*-value of less than 0.05 was considered to be statistically significant. The data were tested against the null hypothesis to confirm the normal distribution. Data analysis was done using GraphPad Prism, version 5.03 (GraphPad Software, San Diego, CA, USA).

## Results

### TfR1 Protein Levels Are Elevated in Cortical Samples of 5xFAD Mice but Remain Unaltered in Isolated Brain Microvessels

**Fig. 1:**
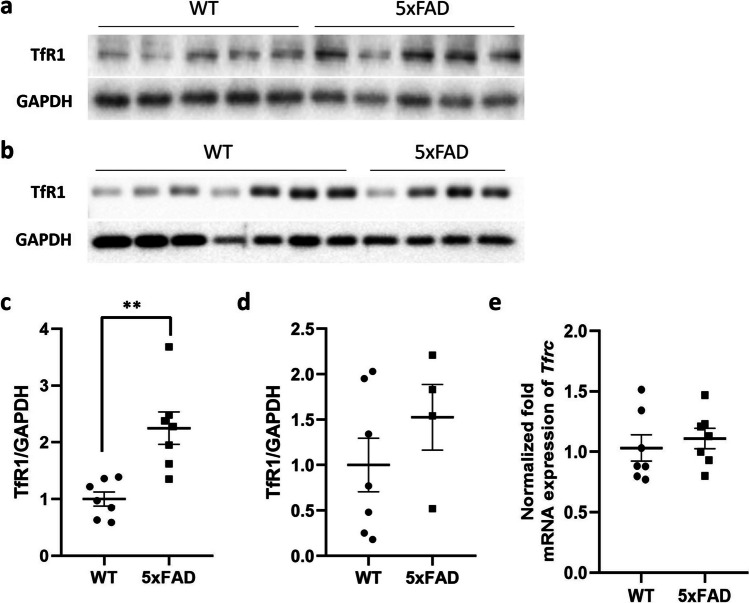
**a**–**d** Western blot and relative densitometry of TfR1 expression in brain cortical samples (**a**, **c**) and in microvessels (**b**, **d**) isolated from 5xFAD transgenic mice versus age-matched wild-type (WT) mice. Data are shown as the ratio between TfR1 and GAPDH as a reference loading control. Each bar represents the mean ± SEM of 7 animals per condition except for 5xFAD microvessels (*n* = 4); **p < 0.01 compared to WT mice, Student’s *t*-test. **e** Gene expression of transferrin receptor (Tfrc) in the brain cortical tissue of 5xFAD and wild-type (WT) mice. The data are presented as mean ± SEM of 7 animals per condition. The gene expression was normalized against the beta-actin (Actb) house-keeping gene

We evaluated whether TfR1 expression levels were different between 5xFAD brain cortical tissue compared to age- and sex-matched WT mice. The results showed significantly higher TfR1 protein expression levels (*p* = 0.0017) in 5xFAD brain cortical tissue compared to WT mice (Fig. 1a, c), while no changes were observed in mRNA expression of *Tfrc* in the same groups (Fig. 1e). In addition, we evaluated TfR1 protein levels in the isolated brain microvessels of 5xFAD mice compared to WT mice, but no statistically significant difference was observed (as shown in Fig. 1b, d).

### Increased TfR1 Expression in Cortical Samples of 5xFAD Mice Is Associated with HIF1A Upregulation

**Fig. 2:**
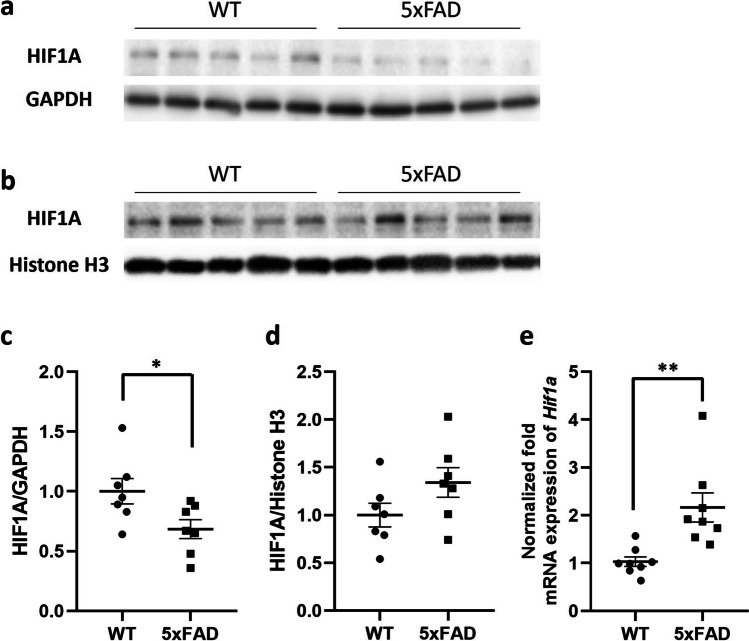
**a**–**d** Western blot and relative densitometry of HIF1A expression in cytosol (**a**, **c**) and nuclei (**b**, **d**) isolated from brain cortical samples of 5xFAD transgenic mice versus age-matched wild-type (WT) mice. Data are shown as the ratio between HIF1A and reference loading control. GAPDH was used as a control for cytosol, and histone H3 for nuclei fraction. Each bar represents the mean ± SEM of 7 animals per condition; *p < 0.05 compared to WT mice, Student’s *t*-test. **e** Gene expression of hypoxia-inducible factor 1α (Hif1a) in the brain cortical tissue of 5xFAD and wild-type (WT) mice. The data are presented as mean ± SEM of 8 animals per condition. The gene expression was normalized against the beta-actin (Actb) house-keeping gene. **p < 0.01 compared to WT mice, Student’s *t*-test

We investigated whether HIF1A was involved in the upregulated TfR1 protein levels in brain cortical samples of 5xFAD mice. Since HIF1A must be translocated from the cytoplasm into the nucleus to exert its function of regulating the expression of the target genes, including the TfR1, we evaluated the expression levels of HIF1A in both nucleic and cytosolic extracts on protein level, as well as in the whole brain cortical tissue in mRNA level. A significant reduction in HIF1A levels was observed in cytosolic extracts of 5xFAD mice compared to WT mice (*p* = 0.033; Fig. 2a, c), accompanied by a parallel increase in HIF1A expression in nuclear extracts of the same mice even if not statistically significant (*p* = 0.055; Fig. 2b, d). In addition, the results showed a statistically significant 2.1-fold increase in the mRNA expression of *Hif1a* in 5xFAD mice compared to WT mice (*p* = 0.0033; Fig. 2e).

### HIF1A Activation Is Associated with Oxidative Stress and Inflammation in 5xFAD Mice

**Fig. 3:**
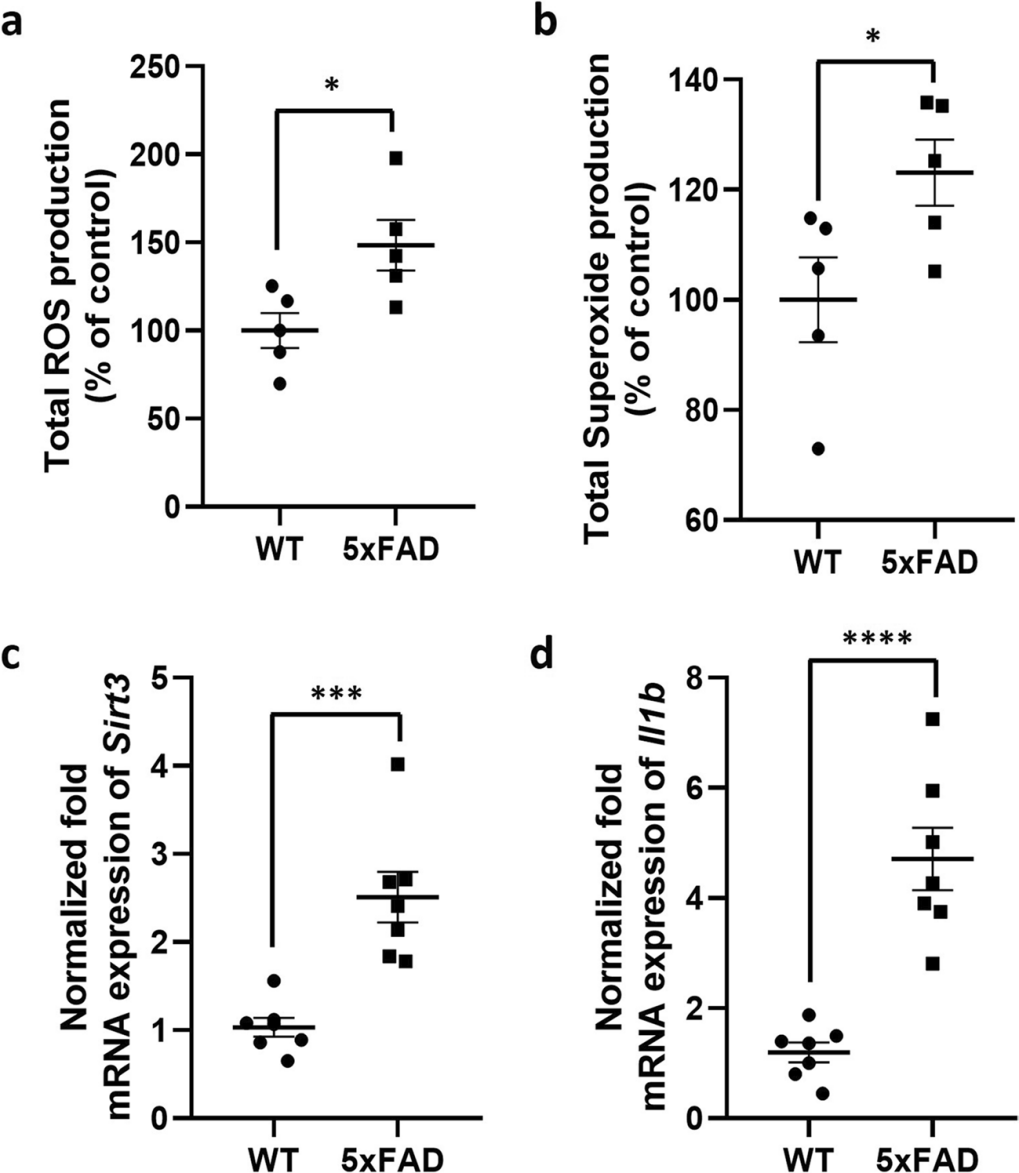
**a**, **b** Total ROS (**a**) and superoxide (**b**) production in the brain cortical tissue of 5xFAD mice and wild-type (WT) mice. The data are presented as mean ± SEM of 5 animals per condition. *p < 0.05 compared to WT mice, Student’s *t*-test. **c**, **d** Gene expression of sirtuin-3 (Sirt3; c) and interleukin-1 beta (Il1b; d) in the brain cortical tissue of 5xFAD and wild-type (WT) mice. The data are present as mean ± SEM of 7 animals per condition. The gene expression was normalized against the beta-actin (Actb) house-keeping gene. ***p < 0.001; ****p < 0.0001 compared to WT mice, Student’s *t*-test

To clarify the effect of the interplay between oxidative stress and inflammation on HIF1A-associated TfR1 expression, we quantified the total ROS and superoxide production, as well as the mRNA expression of sirtuin3 (*Sirt3*) and interleukin-1 beta (*Il1b*) in the brain cortical tissue of 5xFAD and WT mice. The results shown in Fig. 3 demonstrate a statistically significant increase in Total ROS (*p* = 0.024; Fig. 3a) and superoxide (*p* = 0.046; Fig. 3b) production in the brain of 5xFAD mice compared to WT mice. Additionally, a statistically significant increase in the mRNA expression of *Sirt3* and *Il1b* (fold change (FC) = 2.5, *p* = 0.0004; FC = 4.0, *p* < 0,0001, respectively) was observed in 5xFAD mice cortex compared to WT mice (Fig. 3c, d).

### TfR1 Expression Levels Remain Unaltered in Aβ_1-40_- and Aβ_1-42_-Treated hCMEC/D3 Cells

**Fig. 4:**
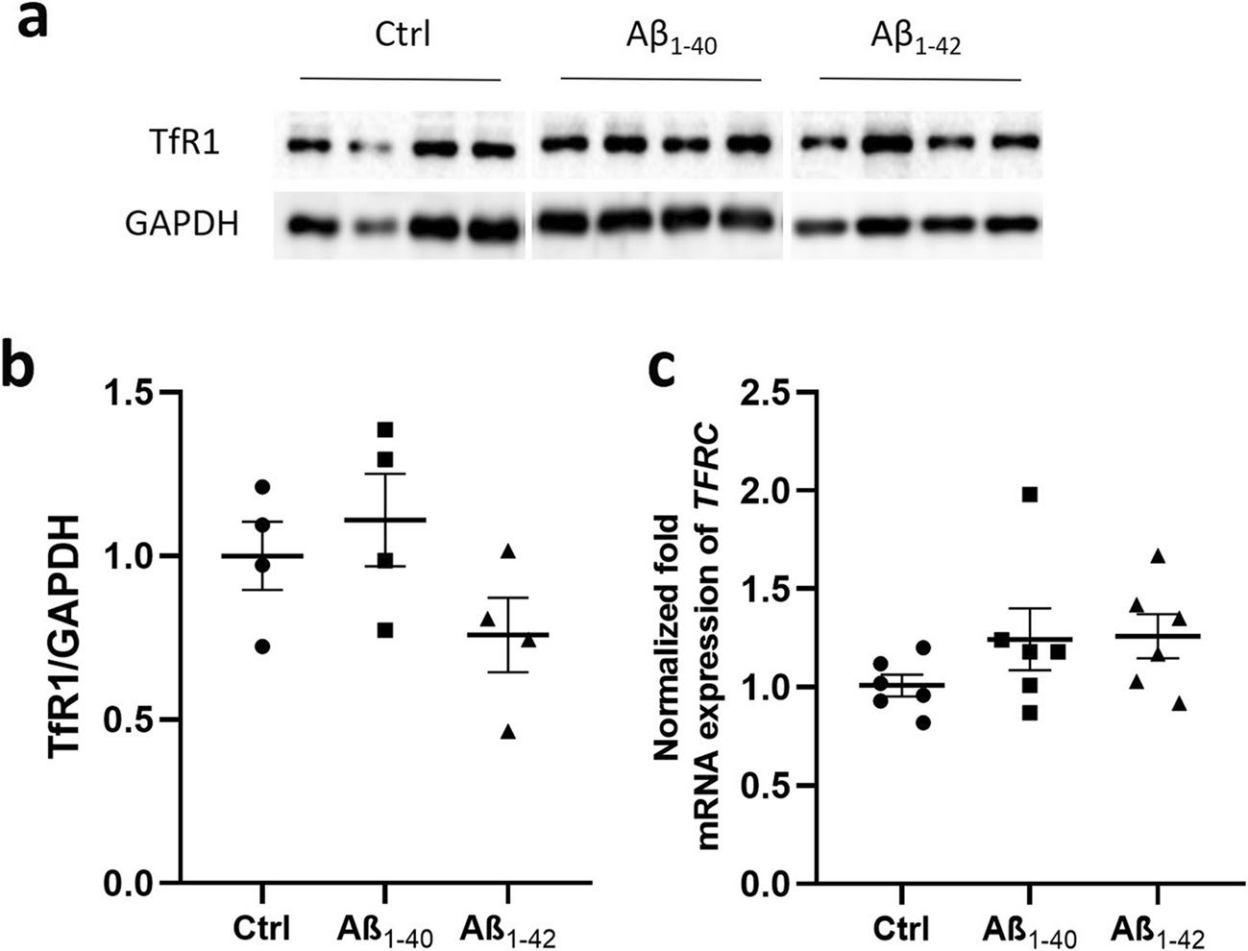
**a**–**b** Western blot and relative densitometry of TfR1 expression in hCMEC/D3 cells treated with 0.1 µM of Aβ1-40 and Aβ1-42 for 48 h compared to untreated cells (Ctrl). Data are shown as the ratio between TfR1 and GAPDH as reference loading control. Each bar represents the mean ± SEM of 4 samples. Student’s *t*-test. **c** Gene expression of transferrin receptor (TFRC) in hCMEC/D3 cells treated with 0.1 µM of Aβ1-40 and Aβ1-42 for 48 h compared to untreated cells (Ctrl). The data are presented as mean ± SEM of 6 samples per condition. The gene expression was normalized against the glyceraldehyde-3-phosphate dehydrogenase (GAPDH) house-keeping gene. Student’s *t*-test

To investigate if the presence of Aβ around brain vasculature is able to induce the changes in TfR1/*TFRC* expression, we treated hCMEC/D3 cells with either 0.1 µM of Aβ_1-40_ or Aβ_1-42_ for 48 h and compared the expression of TfR1/*TFRC* in these cells to untreated control cells. First, we confirmed that Aβ_1-40_ and Aβ_1-42_ at concentration of 0.1 µM did not affect cell viability (Fig. S1). The study showed no changes in TfR1 protein and *TFRC* gene expression in both Aβ_1-40_- (Fig. 4a, c, e) and Aβ_1-42_- (Fig. 4b, d, e) treated hCMEC/D3 cells compared to the control condition (untreated cells).

## Discussion

In the present study, we demonstrated that protein expression of TfR1, the main player involved in the regulation and distribution of iron in the brain, is upregulated in the brain cortex of female 8-month-old 5xFAD mice. The used 5xFAD mice mimic the pathological changes observed in advanced stage of AD, such as Aβ pathology and inflammation as confirmed in the present (Fig. S2) and in our previous study [33]. In addition, our study provided the first evidence that HIF-1 signaling pathway is associated with altered expression of TfR1 in the brain cortical tissue of this mouse model of AD. As iron levels have been found to increase during the disease progression in AD brains as well as in the cortex of the 5xFAD mice and in other mouse models mimicking the advanced stage of AD [19, 44–46], our study provides evidence that TfR1 is likely to play a role in elevated levels of iron in AD. In addition, as it has been reported that altered levels of iron can accentuate toxic Aβ deposition and τ hyperphosphorylation aggregation [15], our findings suggest that TfR1 can play an important role in AD progression via regulating iron levels in the brain.

Although we observed a similar increasing trend in TfR1 expression in the isolated brain microvessels of 5xFAD mice, the changes were not statistically significant. This may be explained by the high variability in TfR1 expression in the isolated brain microvessels between animals and the limited sample size of the 5xFAD group. Future studies with a larger sample size should investigate potential changes in TfR1 expression in the isolated brain microvessels in 5xFAD mice. These discrepancies in changes in expression of TfR1 in the isolated brain microvessels, which mainly represent the changes in endothelial cells of the brain vasculature, and brain cortical tissue, consisting of various brain parenchymal cells, provide evidence that the mechanism of regulation of TfR1 expression in various brain cells is likely to be different. Previously, we reported upregulation of glial fibrillary acidic protein (*Gfap*) expression, a marker of abnormal activation and proliferation of astrocytes, in the same 5xFAD mice that were used in the present study [33]. Thus, one can assume that upregulated TfR1 expression can be a result of increased TfR1 localized to reactive astrocytes.

Previous studies reported ambiguous information on the expression of TfR1 in AD patients and animal models [5, 21, 22, 24]. Our findings of increased TfR1 expression in 5xFAD mice mimicking the advance stage of AD are not in agreement with the hypothesis of Lu et al. (2014), who suggested that TfR1 is upregulated at the early stage of AD as was shown in the cortex and hippocampus of APP/PS1 mice, and decrease with the AD progression [5, 24]. The increased expression of TfR1 in the brain cortex of 5xFAD mice could be one of the factors responsible for the increase in iron content observed in the cortex of the same mouse model at advanced age [46]. Interestingly, in recent study by Belaya et al. (2021), an increase in *Tfrc* gene expression with unaltered protein levels of TfR1 was observed in male 7-month-old 5xFAD mice indicating potential sex- and pathology-dependent changes in this model [47]. Therefore, a characterization of the animal models in terms of TfR1 expression and receptor function is critical during the development of drugs targeting TfR1-related pathways as well as evaluating efficacy of TfR1-mediated drug delivery strategies.

The close relationship between iron, amyloidogenesis, and AD has been confirmed [48, 49]. However, there remain a number of uncertainties about their correlation with the expression of iron transporters and, in particular, with TfR1. Indeed, the majority of AD research has focused on how iron can contribute to Aβ and τ pathology deposition, but it has remained unknown if a dysregulation of iron transporters expression can contribute to Aβ pathology. Here, for the first time, we investigated the effect of Aβ pathology on TfR1 expression changes in an in vitro model represented by hCMEC/D3 cells after Aβ_1-40_ or Aβ_1-42_ treatment. In the present study, no changes in TfR1 protein and in *TFRC* gene expression were detected in hCMEC/D3 cells treated with both Aβ_1-40_ and Aβ_1-42_. Our data supported the preliminary results observed in isolated microvessels and indicated that the Aβ pathology alone was not able to induce TfR1 upregulation in the endothelial cells of the brain vasculature. One can assume that iron dyshomeostasis with TfR1 upregulation observed in 5xFAD mice may facilitate Aβ accumulation, which, in turn, can exacerbate metal dyshomeostasis and TfR1 alterations.

There is growing evidence that TfR gene transcription is regulated by hypoxia and that HIF-1A, the best characterized transcriptional activator of hypoxia-sensitive genes, is the key player in this process [25, 26, 50]. Stable in the cytoplasm, HIF1A can translocate into the nucleus and dimerize with Hypoxia-inducible factor 1-beta (HIF1B). The HIF complex binds to the hypoxia response element (HRE) inducing the transcription of genes relevant to iron metabolism, such as the TfR1 [51, 52]. However, there is a lack of information about the role of HIF-1 signaling in regulation of TfR1 expression in AD. In the present study, we observed a significant increase in the mRNA expression of *Hif1a* in the brain cortical tissue of 5xFAD mice. Moreover, we found a significantly reduced protein expression of HIF1A in cytosolic extracts of the brain cortices in 5xFAD mice compared to WT mice supporting the stabilisation and translocation of HIF1A from the cytosol to the nucleus. In the nuclear extracts from the brain cortices of 5xFAD mice, HIF1A protein expression was not significantly increased, likely due to the nuclear heterodimerization of the HIF1A subunit with the HIF1B subunit for the HIF complex generation. In agreement with the previous studies [25, 26, 50], our data support the hypothesis that the change in TfR1 expression observed in the cortical samples of 5xFAD mice is mediated by HIF-1 signaling pathway.

It has been reported that HIF-1A is typically activated under hypoxic conditions but can also be turned on by non-hypoxic stimuli, including ROS and inflammatory signals [27, 53]. It has been well established that oxidative stress is one of the earliest events that occurs in the pathogenesis of AD. Indeed, increased oxidative stress and redox-active iron have also been confirmed in the brains of patients with mild cognitive impairment, the first symptomatic stage of AD [54]. In the present study in 5xFAD mouse model, we observed an increase in oxidative stress as shown by elevated total ROS and superoxide production. In addition, 5xFAD mice were characterized by inflammation in the brain cortex, which was demonstrated by an increase in the mRNA expression of *Il1b*, the major proinflammatory cytokine, and *Sirt3*, one of the most prominent deacetylases involved in inflammation suppression and in inhibition of oxidative stress [55]. Based on these findings in the studied 5xFAD mice mimicking the advanced stage of AD, we suggest that increased oxidative stress and inflammatory signals are likely to induce the stabilisation and the transactivation of HIF-1A into the nucleus, stimulating HIF-1-mediated TfR1 expression in the brain. This, in turn, can lead to iron dyshomeostasis and exacerbation of Aβ pathology in the brain in AD.

However, some limitations of this study must be acknowledged. One of which is the use of the animals with a pronounced AD pathology, which makes it difficult to evaluate if the observed changes in TfR1 expression precede and contribute to the development of Aβ pathology or are a consequence of the pathological changes occurring in 5xFAD mice by this age. Therefore, future studies should focus on investigating age-dependent changes in TfR1 expression in 5xFAD mice and other animal models of AD. As previously mentioned, our data on the isolated cerebral microvessels of 5xFAD mice were conducted on a limited number of animals. Therefore, further studies with larger sample sizes should be performed to confirm the potential role of brain parenchymal cells in TfR1 expression changes. In addition, the expression analysis of TfR1 should be complemented with functional tests of the receptor in 5xFAD mice. Finally, further investigation is necessary to clarify the effect of AD pathology on the modulation of HIF1A and to elucidate mechanisms involved in the regulation of TfR1 via HIF-1 signaling pathway.

Overall, the present study provides evidence that, in AD, under hypoxic conditions and non-hypoxic stimuli, such as oxidative stress and inflammation, activation of HIF-1-signaling can lead to TfR1 upregulation, which can result in iron dyshomeostasis in the brain, which, in turn, can contribute to AD pathology. Therefore, modulation of TfR1 expression via targeting HIF-1-signaling pathway and related mechanisms can be a promising therapeutic approach for treatment of AD.

## Conclusion

In the present work, we characterized a commonly used model of familial AD, female 5xFAD mice mimicking advanced stages of AD pathology, in terms of changes in the brain expression of TfR1. Here, we observed various alterations in TfR1 expression, such as upregulation of TfR1 in the brain cortical tissue and no effect on expression in the isolated brain microvessels of 5xFAD mice indicating cell-specific changes in expression of TfR1 in advanced stages of AD. In addition, using an in vitro model, such as the human immortalized brain endothelial cells hCMEC/D3, we demonstrated for the first time that Aβ pathology is not likely affecting the expression of TfR1 in the brain endothelial cells. Furthermore, the present study provides evidence that the upregulation of TfR1 expression in the brain cortical tissue of 5xFAD mice is associated with activation of HIF-1 signaling pathway as well as oxidative stress and inflammation. Considering a significant role of TfR1 in brain iron transport and drug delivery to the CNS, this study provides important information on changes in TfR1 expression and involvement of HIF-1 signaling pathway in these changes in 5xFAD mice. In addition, the findings of the study demonstrated that modulating TfR1 expression via targeting HIF-1 signaling pathway may be a novel pharmacological intervention for the treatment of AD. Overall, the study provides important information for developing drugs targeting HIF-1 signaling pathway in AD, as well as testing drugs and evaluating the efficacy of the TfR1-mediated drug delivery systems in the 5xFAD model.

### Supplementary Information

Below is the link to the electronic supplementary material.

Supplementary file1 (DOCX 87 KB)

## Data Availability

The datasets generated during and/or analyzed during the current study are available from the corresponding author on reasonable request.
