# Supplementary material for: Increased Expression of Transferrin Receptor 1 in the Brain Cortex of 5xFAD Mouse Model of Alzheimer’s Disease Is Associated with Activation of HIF-1 Signaling Pathway
Source: Mol Neurobiol. 2024 Feb 1;61(9):6383–94. doi: 10.1007/s12035-024-03990-3 (PMC11339108; doi:10.1007/s12035-024-03990-3)
Supplement: Supplementary file 1 — Supplementary file1 (DOCX 87 KB) [file 12035_2024_3990_MOESM1_ESM.docx]

**Supporting information**

**Increased expression of transferrin receptor 1 in the brain cortex of 5xFAD mouse model of Alzheimer’s disease is associated with activation of HIF-1 signalling pathway**

*Sabrina Petralla^a^, Liudmila Saveleva^b^, Katja M. Kanninen^b^, Julia S. Oster^a^, Maria Panayotova^a^, Gert Fricker**^a^, Elena Puris^a*^*

*^a^Institute of Pharmacy and Molecular Biotechnology, Ruprecht-Karls-University, Im Neuenheimer Feld 329, 69120 Heidelberg, Germany*

*^b^A.I. Virtanen Institute for Molecular Sciences, University of Eastern Finland, P.O. Box 1627, 70211 Kuopio, Finland*

***Corresponding author:** Elena Puris, Institute of Pharmacy and Molecular Biotechnology, Ruprecht-Karls-University, Im Neuenheimer Feld 329, 69120 Heidelberg, Germany;

phone: +(358)449789164; email: [elena.puris@uni-heidelberg.de](mailto:elena.puris@uni-heidelberg.de)

ORCID ID: https://orcid.org/0000-0002-1769-389X

***Table S1.*** *Mouse primer sequences for SYBR Green qRT-PCR*

| **Gene** | **Forward Primer** | **Reverse Primer** |
| --- | --- | --- |
| *Tfrc* | 5′-GGAGGACGCGCTAGTGTTC-3′ | 5′-TCCAACAAGGAACATGATGC-3′ |
| *Hif1a* | 5′-CCTGCACTGAATCAAGAGGTTGC-3′ | 5′-CCATCAGAAGGACTTGCTGGCT-3′ |
| *Sirt3* | 5′-GCTACATGCACGGTCTGTCGAA-3′ | 5′-CAATGTCGGGTTTCACAACGCC-3′ |
| *Il1b* | 5′-GCAACTGTTCCTGAACTCAACT-3′ | 5′-ATCTTTTGGGGTCCGTCAACT-3′ |
| *Actb* | 5′-AAGTCCCTCACCCTCCCAAAAG-3′ | 5′-ACACAGAAGCAATGCTGTCACC-3′ |

***Table S2.*** *Human primer sequences for SYBR Green qRT-PCR*

| **Gene** | **Forward Primer** | **Reverse Primer** |
| --- | --- | --- |
| *TFRC* | 5′-GCACAGCTCTCCTATTGAAAC-3′ | 5′-GGTATCCCTCTAGCCATTCAG-3′ |
| *GAPDH* | 5′-CCATCACCATCTTCCAGGAGCGA-3′ | 5′-GGATGACCTTGCCCACAGCCTTG-3′ |

**
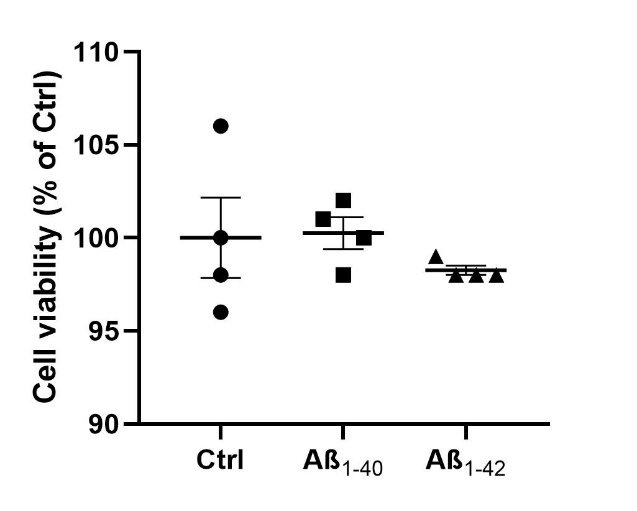
**

***Fig. S1.*** *Effect of* *0.1 µM of Aβ_1-40_ and Aβ_1-42_ on hCMEC/D3 cell viability after 48h treatment compared to untreated cells (Ctrl). Results are the mean ± SEM of 4 samples per condition.*

**
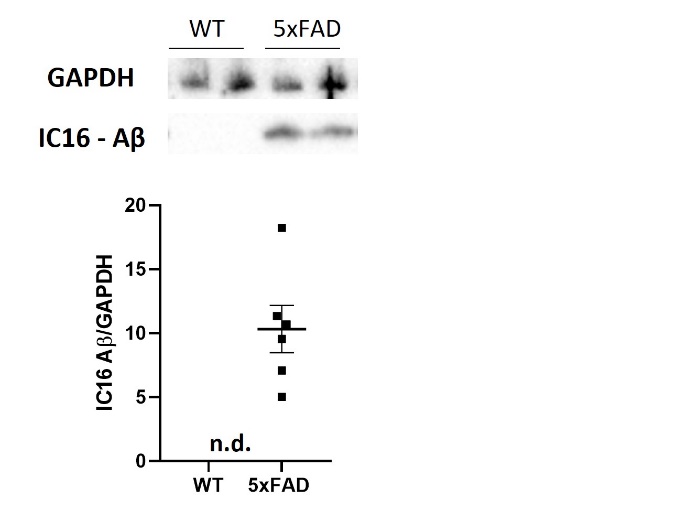
**

***Fig. S2.*** *Western Blot and relative densitometry of Aβ IC16 expression in brain cortical samples of 5xFAD transgenic mice versus age-matched wild-type (WT) mice. Data are shown as the ratio between Aβ IC16 and GAPDH as reference loading control. Bar represents the mean ± SEM of 6 animals for 5xFAD mice and 2 animals for WT mice; n.d. = not detectable.*
